# Transplantation of murine neonatal cardiac macrophage improves adult cardiac repair

**DOI:** 10.1038/s41423-020-0371-5

**Published:** 2020-02-13

**Authors:** Yandong Li, Haotong Li, Jianqiu Pei, Shengshou Hu, Yu Nie

**Affiliations:** State Key Laboratory of Cardiovascular Disease, Fuwai Hospital, National Center for Cardiovascular Disease, Chinese Academy of Medical Sciences and Peking Union Medical College, Beijing, 100037 China

**Keywords:** Cell biology, Immunology

Heart regeneration is a promising strategy to prevent cardiac injury and protect against heart failure.^1–3^ Distinguishing from adult mammals, neonatal mouse heart can regenerate completely after cardiac injury, such as apical resection (AR) or myocardial infarction (MI). Our previous study reveals that acute inflammation is essential for initiation of neonatal mouse heart regeneration,^4^ providing an insight to understand the role of immune system in cardiac repair. Macrophage is a prominent inflammatory cell in injured heart during acute immune response;^5^ here we revealed that genetically deletion of CD11b-positive macrophages in *CD11b*^*DTR*^ mice with diphtheria toxin (DT) administration led the suspense of neonatal heart regeneration after AR injury. We transplanted cardiac macrophages sorted from AR-injured neonatal mouse hearts into MI-injured adult mice and found that transfusion of neonatal mouse cardiac macrophages facilitated cardiac repair and enhanced cardiomyocyte proliferation effectively, which served a potential intervention to improve prognosis of patients suffering cardiac diseases.

In 2015, we reported that zymosan A, inducer of the innate immune response, could promote cardiomyocyte proliferation effectively by stimulating acute inflammation when we intramyocardially microinjected zymosan into neonatal mouse hearts with the thinnest needle injector.^4^ A recent study claims that intra-cardiac injection of both zymosan A and cell debris, freeze/thaw-killed cells, can promote cardiac repair and pumping function recovery after ischemia/reperfusion (I/R) injury via stimulating acute immune response and recruitment of macrophages, instead of stem cells differentiating into cardiomyocytes,^6^ confirming the constructive roles of acute inflammation and macrophages in cardiac repair. Noteworthy, compared to 1-day aged mice, with lost cardiac regenerative capacity during postnatal development, the type of cardiac macrophages in 14-day aged mouse myocardium was transformed,^7^ indicating that cardiac macrophages in 1-day-old mouse heart might be more effective to promote cardiomyocyte proliferation. Thus we speculated that transplantation of neonatal cardiac macrophages could improve cardiac repair in MI-injured adult mice.

**Fig. 1:**
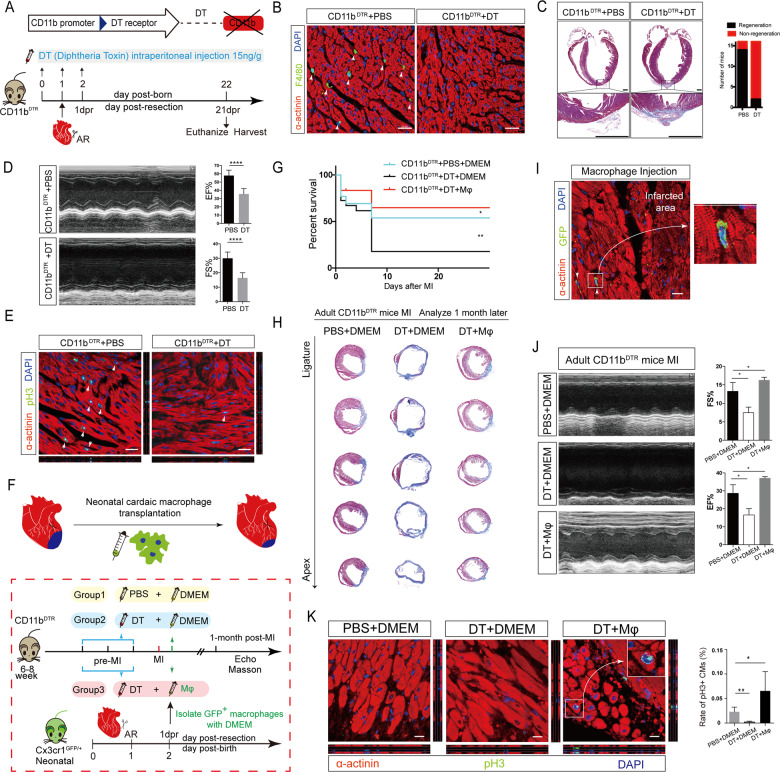
Implication of neonatal cardiac macrophage ameliorates cardiac repair in myocardial infarction injured adult mice. **a** Experimental strategy for deleting macrophages using DT in *CD11b*^*DTR*^ mice, followed by apical resection at day 1. DT is administrated from day 0 to 2 for consecutive three days. Mice are sacrificed at 21 days after the operation. **b** IF shows that macrophages infiltration after heart injury is suspended after DT administration in *CD11b*^*DTR*^ mice. **c** Representative images for Masson’s trichrome staining show that heart cannot regenerate but with fibrosis formation in the macrophage-deleted group and DT administration leads to lower quantities of regenerative mice (*n*=16 for each group). **d** Representative images of echocardiography in macrophage-deleted mice after apical resection operation. Statistical analysis shows the lower EF% and FS% in macrophage-deleted mice. Data are mean ± SEM. ****P<0.0001. **e** IF shows that macrophages deletion suspends cardiomyocytes proliferation identified by decreased pH3 positive α-actinin. **f** Scheme of deletion of macrophages in adult mice, transplantation of neonatal cardiac macrophages into adult injured heart as a treatment therapy. Strategy of injecting GFP positive macrophages isolated from *Cx3cr1*^*GFP/+*^mice to *CD11b*^*DTR*^ adult mice following myocardial infarction operation. **g** Kaplan-Meier survival curve in adult mice following myocardial injection induction shows that macrophages transplantation has the highest survival rate. Data are mean ± SEM. *P<0.05, **P<0.01. **h** Representative images for Masson’s trichrome staining shows macrophages injection has the smallest infarcted area in adult CD11bDTR mice (PBS+DMEM, *n*=12; DT+DMEM, *n*=14; DT+Macrophage *n*=14). **i** Neonatal cardiac macrophages (GFP positive) can be detected in adult mouse heart after injection. **j** Representative images of echocardiography in macrophage-injected mice after myocardial infarction operation. Statistical analysis shows the highest EF% and FS% in macrophage-injected adult mice. Data are mean±SEM. *P<0.05. **k** If shows that macrophages transplantation can induced adult cardiomyocytes proliferation (*n*=6 for each group). Data are mean±SEM. *P<0.05, **P<0.01

In this study, AR was performed on 1-day-old *Cd11b*^*DTR*^ mice, which express a DT-inducible system, controlled by the human Cd11b promoter, enabling efficient depletion of macrophages (Fig. 1a). Immunostaining showed that macrophages were abundantly infiltrated into myocardium at 1 day post-resection (dpr) in *Cd11b*^*DTR*^ mice without DT injection (Fig. 1b). And macrophages were undetectable in DT-injected *Cd11b*^*DTR*^ mice at 1 dpr (Fig. 1b). Masson’s trichrome staining showed that fiber scar formed in AR injured myocardium without heart regeneration at 21 dpr once macrophages were abolished, while the heart of *Cd11b*^*DTR*^ mice without DT treatment could regenerate completely (Fig. 1c). Echocardiography revealed that macrophage deletion led to significantly progressive cardiac function deterioration (decreased ejection fraction percentage and fractional shortening percentage, *n* = 16, *p* < 0.0001) (Fig. 1d). We also found that pH3-positive proliferative cardiomyocytes were decreased significantly in macrophage-deficient mouse myocardium at 7 dpr (Fig. 1e). Our results provided the essential role of CD11b-postive macrophages in heart regeneration with *Cd11b*^*DTR*^ transgenic mice.

Compared with adult *Cd11b*^*DTR*^ mice without DT treatment, 83.3% macrophage-deficient mice (adult *Cd11b*^*DTR*^ mice with DT treatment) were dead in 7 days after MI (Fig. 1g), with larger infarcted area and worse function (Fig. 1h, j). To investigate whether neonatal cardiac macrophages could promote cardiac repair in adults, we sorted AR-injury-recruited neonatal cardiac macrophages at 1 dpr by FACS Aria II from the hearts of *Cx3cr1*^*GFP/+*^ mice (Fig. 1f), which expressed enhanced green fluorescent protein (GFP) under the endogenous Cx3cr1 locus to identify cardiac macrophages. Then we injected neonatal cardiac macrophages suspending in 200 μl Dulbecco’s Modified Eagle Medium (DMEM) into adult *Cd11b*^*DTR*^ mice within 6 h after MI by tail intravenous injection (Fig. 1f). The GFP-positive macrophages could be detected in adult *Cd11b*^*DTR*^ mouse myocardium 1 day after macrophage transplantation (Fig. 1i). Our results showed that implantation of neonatal cardiac macrophage improved cardiac repair and heart function significantly in MI-injured macrophage-deficient *Cd11b*^*DTR*^ mice, even comparing with *Cd11b*^*DTR*^ mice without DT administration (Fig. 1g, h, j).

To explore whether neonatal cardiac macrophage implantation ameliorates MI-injured adult mouse cardiac repair *via* stimulating cardiomyocyte proliferation, we evaluated the ratio of proliferative cardiomyocytes by detecting pH3 and α-actinin double-positve cellsat 7 days after macrophage transplantation (Fig. 1f). Our results revealed that the proliferative cardiomyocytes were increased significantly in MI-injured myocardium with transfusion of neonatal cardiac macrophages and hard to detect both in DT-treated and untreated MI-injured *CD11b*^*DTR*^ mice (Fig. 1k), indicating that transplantation neonatal cardiac macrophage played a cardiomyocyte pro-proliferative role in adult cardiac repair.

Collectively, employing *CD11b*^*DTR*^ genetically macrophage-deficient mice, we illustrated that macrophage recruitment is a critical response for neonatal heart regeneration after cardiac injury. Enhancement of cardiomyocyte proliferation is undetectable in cardiac repair improved by stimulating immune response with zymosan, cell therapy, or cell debris.^6^ Distinctively, our results showed that transplantation of neonatal cardiac macrophage could improve MI-injured adult cardiac repair via inducing cardiomyocyte proliferation. Our results attest that macrophages from neonatal hearts have regenerative function, providing a potential strategy to promote cardiac repair.
